# Metalloproteases Affecting Blood Coagulation, Fibrinolysis and Platelet Aggregation from Snake Venoms: Definition and Nomenclature of Interaction Sites

**DOI:** 10.3390/toxins8100284

**Published:** 2016-09-29

**Authors:** R. Manjunatha Kini, Cho Yeow Koh

**Affiliations:** Protein Science Laboratory, Department of Biological Sciences, Faculty of Science, 14 Science Drive 4, National University of Singapore, Singapore 117543, Singapore; choyeow@nus.edu.sg

**Keywords:** procoagulant, anticoagulant, factor X activator, prothrombin activator, platelet aggregation, fibrinolytic, exosites in enzymes, allosteric sites

## Abstract

Snake venom metalloproteases, in addition to their contribution to the digestion of the prey, affect various physiological functions by cleaving specific proteins. They exhibit their activities through activation of zymogens of coagulation factors, and precursors of integrins or receptors. Based on their structure–function relationships and mechanism of action, we have defined classification and nomenclature of functional sites of proteases. These metalloproteases are useful as research tools and in diagnosis and treatment of various thrombotic and hemostatic conditions. They also contribute to our understanding of molecular details in the activation of specific factors involved in coagulation, platelet aggregation and matrix biology. This review provides a ready reference for metalloproteases that interfere in blood coagulation, fibrinolysis and platelet aggregation.

## 1. Introduction

Snake venoms are cocktails of pharmacologically active proteins and peptides. They are used as offensive weapons in immobilizing, killing and digesting the preys [1,2]. Some of these toxins exhibit various enzymatic activities, whereas others are nonenzymatic proteins. Most enzymes found in snake venoms are hydrolases that breakdown biological molecules including proteins, nucleic acids and phospholipids. In addition to their contribution to the digestion of the prey, a number of these hydrolases exhibit specific pharmacological effects. Snake venoms, particularly crotalid and viperid venoms, are rich sources of metalloproteases and serine proteases.

**Figure 1:**
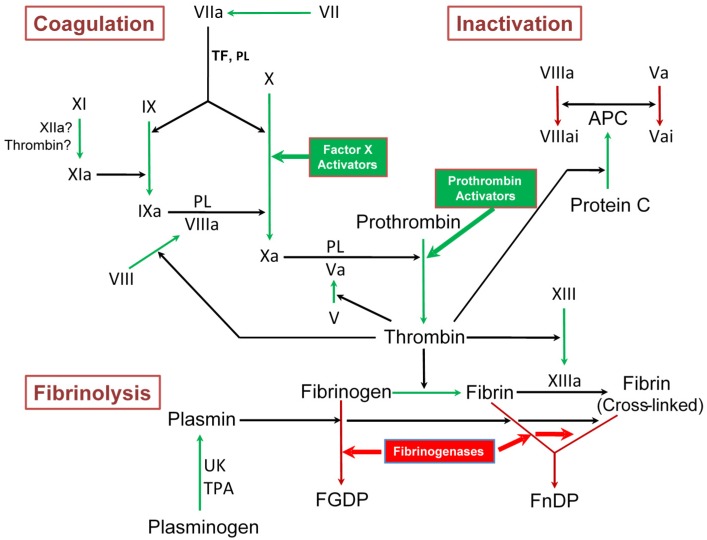
Snake venom metalloproteases affecting blood coagulation. Proteinases interfere by proteolysis of specific factors (thick arrow heads). Green boxes, procoagulant SVMPs; red box, fibrinogenases that cleave fibrinogen and fibrin; APC, activated protein C; FGDP, fibrinogen degradation products; FnDP, fibrin degradation products; PL, phospholipids; TF, tissue factor; TPA, tissue plasminogen activator; UK, urokinase.

**Figure 2:**
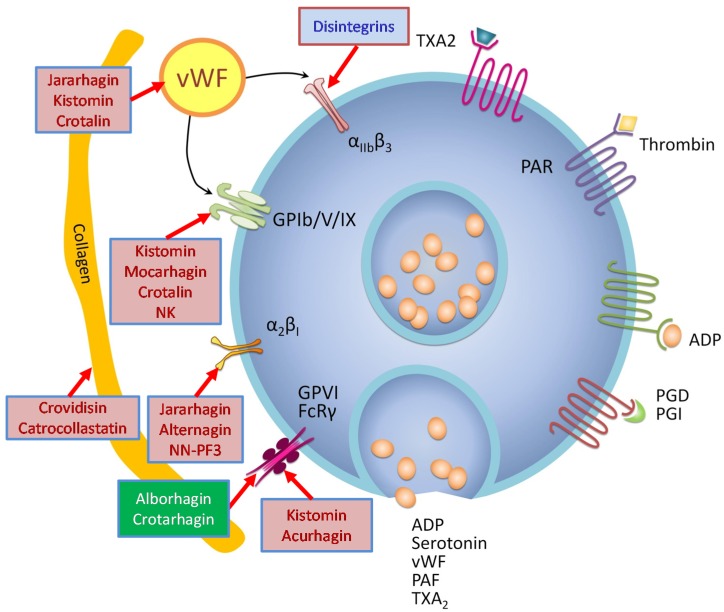
Snake venom metalloproteases affecting platelet aggregation. Proteinases that induce or inhibit platelet aggregation are shown in green or red boxes, respectively; Disintegrins that inhibit platelet aggregation are shown in blue box; PAF, platelet activating factor; PAR, protease activated receptor; PGD, prostaglandin D; PGI, prostaglandin I; TXA2, thromboxane A_2_.

Snake venom metalloproteases (SVMPs) are Zn^2+^-dependent, endoproteolytic enzymes that are classified into three different classes: P-I, P-II and P-III [3,4]. They are closely related to ADAM (a disintegrin and metalloprotease) family proteins and are included in the M12B clan [5]. SVMPs selectively cleave a small number of key proteins in the blood coagulation cascade and in platelet aggregation. Such limited proteolysis leads to either activation or inactivation of the protein involved in these processes, thus resulting in interference in blood coagulation and platelet aggregation (Figure 1 and Figure 2). This review provides an overview on a number of metalloproteases that interfere in blood coagulation, fibrinolysis and platelet aggregation.

## 2. Procoagulant Proteases

Blood coagulation factors circulate as zymogens and they get activated through limited proteolytic cleavage during the breach of the blood vessel in a sequential manner leading to formation of fibrin clot that stops the blood leakage. All procoagulants from snake venoms characterized to date are proteases; they activate a zymogen of specific coagulation factors in the coagulation cascade and hasten clot formation. Unlike snake venom serine proteases, which activate various zymogens in the coagulation cascade (for reviews, see [6,7]), SVMPs activate only two key coagulation factors, factor X (FX) and prothrombin to exhibit their procoagulant effects.

### 2.1. Factor X Activators

Venoms from Viperidae, Crotalidae and Elapidae contain a variety of proteases capable of activating factor X (for reviews, see [8,9]). They are either metalloproteases or serine proteases. In general, metalloprotease FX activators are found in Viperidae and Crotalidae venoms [10,11,12], while serine protease FX activators are found in Elapidae venoms [13,14,15]. All the metalloprotease FX activators have two subunits held together by inter-subunit disulfide linkage; larger subunit is a P-III metalloprotease whereas the smaller subunit is a snaclec (snake C-type lectin-related proteins) with two chains covalently linked by an inter-chain disulfide bond. FX activator from Russell’s viper (*Daboia russelli*) venom (RVV-X) is the well-characterized protein (for details, see [8]). As with other P-III enzymes, RVV-X possesses metalloprotease (M), disintegrin-like (D) and cysteine-rich (C) domains. The smaller subunit is a typical C-type lectin-related dimer and contributes to FX selectivity by binding to the γ-carboxy glutamate residues containing Gla domain of FX. Similar to physiological activators, intrinsic tenase (FIXa-FVIIIa) and extrinsic tenase (FVIIa-tissue factor) complexes, RVV-X activates FX by a proteolytic cleavage of Arg152-Ile153 bond resulting in the release of a 52-residue activation peptide and the activated FXaα [16,17]. *Bothrops atrox* activators, however, produce two other cleavages: one near the *N*-terminal end of the heavy chain of FX, generating FXμ, and a second one located at one extremity of the heavy chain of FXaα, generating FXaν [12].

**Figure 3:**
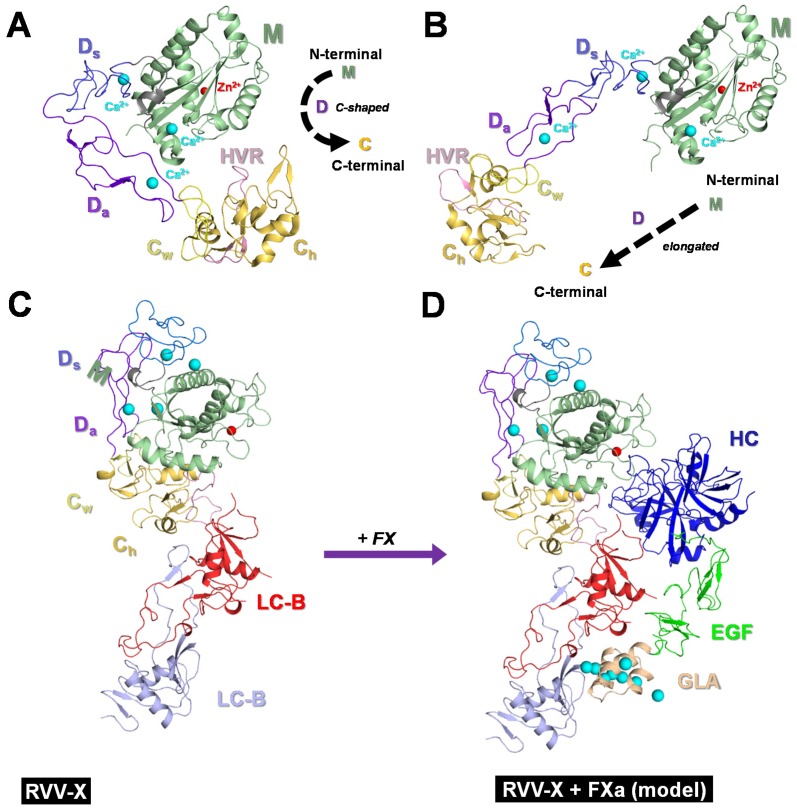
Structure of P-III snake venom metalloproteases. All proteins are shown as ribbon structures. Zn^2+^ and Ca^2+^ ions are shown as red and light blue spheres, respectively. Subdomains and segments are colored and named. (**A**) Catrocollastatin, an inhibitor of collagen-induced platelet aggregation prothrombin activator and a P-III SVMP, showing M, D and C domains, which form a C-shaped configuration (*inset*). (**B**) Kaouthiagin-like protease, in contrast exhibits straight configuration. The presence of “hinges” between the domains help P-III SVMPs to “open” and exhibit straighter configuration. (**C**) Ribbon structure of RVV-X, a P-IIId SVMP. Carinactivase and mutactivase, prothrombin activators, also belong to this class. (**D**) Docking model (prepared by Soichi Takeda) depicting the structural basis of FX activation.

Structural studies of RVV-X and other related P-III enzymes [18,19,20,21,22,23] help elucidate their structure–function relationship. The three domains of P-III SVMPs are arranged into a C-shaped configuration, with the *N*-terminal M domain interacting with C-terminal C domain (Figure 3A). One of the exceptions is kaouthiagin-like protease from *Naja atra*, which adopts a more elongated conformation due to the absence of a 17-residue segment and to a different disulfide bond pattern in the D domain [22] (Figure 3B). Other than variations in the peripheral loops, the structures of M domain among P-III [18,19,20,21,22,23], P-I [24,25,26,27,28,29,30,31,32,33,34] and P-II [35] enzymes are similar. M domains are folded as a five-stranded β-sheet interspersed with five α-helices into two subdomains flanking the catalytic cleft in which a zinc ion is localized. The conserved Zn^2+^-binding H*E*XXHXXGXXHD motif is located at the bottom of the catalytic cleft. The catalytic Zn^2+^ ion is coordinated by the Nε atoms of three His side chains within the consensus motif (underlined) in addition to a solvent water molecule, which in turn is bound to the conserved Glu (italic). The identity of fourth ligand as water is ascertained by quantum mechanical and molecular mechanical simulations [36]. The D domain has two sub-domains named the “shoulder” (D_s_) and the “arm” (D_a_) (Figure 3). The bound Ca^2+^ ions and disulfide bonds in this domain are essential for the rigidity of the C-shaped since it lacks other secondary structural elements [37]. The D_a_ subdomain folds similar to disintegrin [38] with some variations in the RGD-containing disintegrin (D)-loop and the C-terminal region. Although the D-loop of disintegrin is thought to be involved in integrin-binding, it is not accessible for interaction in P-III enzymes as it packs against the C domain. The C domain of P-III SVMPs can be divided into two subdomains, the “wrist” (C_W_) at the N-terminal, and the “hand” (C_h_) towards the C-terminal. The C_w_ subdomain extends from D_s_ and D_a_ subdomains to form the C-shaped arm structure while the C_h_ subdomain forms a separated core of made of a unique α/β -fold structure (Figure 3). Within the C_h_ subdomain, a hyper-variable region (HVR) can be identified and may function in specific protein–protein interactions [18].

P-IIId SVMPs is a subgroup that has additional subunits forming larger complexes. For example, RVV-X is a P-IIId complex [38] consisting of an MDC-containing heavy chain and two light chains of snaclec (Figure 3C). It has a hook-spanner-wrench-like architecture, in which the MD domains of the heavy chain resemble a hook, and the remainder of the molecule constitutes a handle [19]. A disulfide bridge between the Cys389 of heavy chain and Cys133 of light chain A links the two chains. Multiple hydrophobic interactions and hydrogen bonds further stabilize the interface. Light chains A and B are linked via a disulfide bond between Cys79 and Cys77 of the respective chains. The dimeric interface formed by the two snaclecs light chains is a concave structure similar to the ligand-binding site of factor IX/X binding protein [39]. This concave surface is likely to function as an exosite that binds to the gamma-carboxyglutamic acid-rich (Gla) domain of FX in the presence of Ca^2+^ [19]. A docking model indicates that the C_h_/light chain portion may act as a scaffold to accommodate the elongated FX molecule. Ca^2+^ is likely to induce conformational changes in the Gla domain of FX, which might be necessary for the RVV-X recognition [17], consistent with the original proposal [8]. RVV-X is an example of venom complex that has evolved to target specific proteins in the blood coagulation cascade and to cause immediate toxicity to the vertebrate prey by coagulating its blood.

### 2.2. Prothrombin Activators

A large number of snake species contain prothrombin activators in their venoms (for an inventory, see [40], and for reviews, see [41,42,43,44,45,46]). Based on their structural properties, functional characteristics and cofactor requirements, they have been categorized into four groups [40,47,48]. Groups A and B prothrombin activators are metalloproteases and they convert prothrombin to meizothrombin. In contrast, groups C and D prothrombin activators are serine proteases and they convert prothrombin to mature thrombin. Here I will discuss some of the salient features of only groups A and B prothrombin activators. For more details, readers are advised to read recent reviews on prothrombin activators [44,45,46].

#### 2.2.1. Group A Prothrombin Activators

These metalloproteases efficiently activate prothrombin without the requirement of any cofactors, such as Ca^2+^ ions, phospholipids or FVa [40,41]. They are found in several viper venoms and resistant to the natural endogenous coagulation inhibitors, such as serpins and antithrombin III [47]. They probably play the role of toxins in the venom. The best characterized Group A activator is ecarin, isolated from the venom of the saw-scale viper *Echis carinatus* [49]. The mature protein is a metalloprotease with 426 amino acids and shares 64% identity with the heavy chain of RVV-X [50]. Ecarin is also a P-III enzyme with MDC domains. In the disintegrin-like domain, the RGD tripeptide sequence is replaced by RDD sequence. Consequently, ecarin has no inhibitory effect on platelet aggregation. Ecarin is a highly efficient enzyme with a low Km for prothrombin and a high kcat. It cleaves the Arg_320_–Ile_321_ bond in prothrombin and produces meizothrombin. Meizothrombin is ultimately converted to α-thrombin by autolysis. Ecarin can also activate descarboxyprothrombin that accumulates in plasma during warfarin therapy. Other prothrombin activators in this class [40,41], for example, those isolated from the *Bothrops* species [51], also have similar properties. In contrast, serine proteases that activate prothrombin (groups C and D) cleave at both Arg_271_–Thr_272_ and Arg_320_–Ile_321_ bonds of prothrombin [52,53,54,55], converting it to mature thrombin. Structural details of other Group A prothrombin activators are not available.

#### 2.2.2. Group B Prothrombin Activators

In 1996, Yamada et al. [47] isolated and characterized carinactivase-1, another prothrombin activator from *E. carinatus* venom. In contrast to ecarin and other Group A prothrombin activators, this proteinase activity was Ca^2+^-dependent. Similar to RVV-X, carinactivase-1 consists of two subunits held covalently through a disulfide bond: a 62 kDa P-III metalloprotease and a 25 kDa snaclec dimer linked by disulfide bridge. The snaclec subunit is homologous to the factor IX/X-binding protein from *Trimeresurus flavoviridis* venom [8,56]. Carinactivase-1 required millimolar concentrations of Ca^2+^ for its activity and had virtually no activity in the absence of Ca^2+^ ions. The light chains contribute to the specificity as well as Ca^2+^ dependency of Carinactivase-1. Therefore, unlike ecarin, Carinactivase-1 does not activate prothrombin derivatives, prethrombin-1 and descarboxyprothrombin, in which Ca^2+^-binding has been perturbed. Based on this property, Yamada and Morita [57] developed a chromogenic assay for normal prothrombin in the plasma of warfarin-treated individuals. Functionally, the metalloprotease subunit by itself is similar to ecarin: it no longer requires Ca^2+^ for activity. Reconstitution of the snaclec subunit restores Ca^2+^ dependence. Prothrombin activation by carinactivase-1 is inhibited by prothrombin fragment 1, and the isolated snaclec subunit is capable of binding to fragment 1 in the presence of Ca^2+^ ions. Hence this protein recognizes the Ca^2+^-bound conformation of the Gla domain in prothrombin via the 25 kDa regulatory subunit, and the subsequent conversion of prothrombin is catalyzed by the 62-kDa catalytic subunit. Subsequently, another prothrombin activator multactivase in *Echis multisquamatus* venom, which had very similar properties to carinactivase-1 was characterized [58]. Similar to Group A prothrombin activators, these enzymes also produce meizothrombin.

## 3. Fibrinolytic Enzymes

Fibrinogen is cleaved by both venom serine proteases and metalloproteases. Interestingly, serine proteases cleave the N-terminal end of the Aα or Bβ chains of fibrinogen releasing fibrinopeptide A or B, respectively, unlike thrombin, which releases both peptides [59,60]. These thrombin-like enzymes (TLEs) were isolated and characterized from venoms of pit vipers (*Agkistrodon*, *Bothrops*, *Lachesis* and *Trimeresurus*), true vipers (*Bitis* and *Cerastes*) and colubrids, *Dispholidus typus* (for an inventory and reviews, see [60,61,62]). Although classical serine protease inhibitors inhibit TLEs, most are not inhibited by thrombin inhibitors like antithrombin III and hirudin [59,60,63]. TLEs usually form friable and translucent clots presumably due to lack of crosslinking of fibrin by FXIIIa. In contrast, SVMPs selectively cleave the Aα chain of fibrinogen but not cleave Bβ and γ chains and thus classified as α-fibrinogenases [64,65,66,67,68,69,70]. They cleave at the C-terminal end of the Aα chain produce truncated fibrinogen, which is unable to form a stable fibrin clot, and thus inhibit blood coagulation. These SVMPs belong to all three classes, P-I, P-II and P-III. Unlike TLEs, these SVMPs also exhibit fibrinolytic activity. Thus, they may have clinical applications in the treatment of occlusive thrombi [71,72].

## 4. Platelet Aggregation Antagonists

Some α-fibrinogenases, described above, inhibit platelet aggregation [73,74]. Because of their ability to degrade fibrinogen, the antiplatelet effects of fibrinolytic enzymes were suggested to be caused by the formation of inhibitory fibrinogen degradation products [73,75,76]. Subsequent studies, however, showed that the degradation products of fibrinogen produced by either the α-fibrinogenase from *A. rhodostoma* venom or by plasmin do not show antiplatelet effects comparable to the protease [74,77]. Thus, the α-fibrinogenase was proposed to inhibit aggregation by elimination of the intact form of the adhesive molecule fibrinogen [74]. Interestingly, only a small number of but not all fibrinogenases inhibit platelet aggregation. Thus, the role of fibrinogen degradation in the inhibition of platelet aggregation by α-fibrinogenases was questionable. Our studies using F1-proteinase, an α-fibrinogenase from *Naja nigricollis* venom, showed that the degradation products of fibrinogen formed by this protease failed to inhibit platelet aggregation [78]. This SVMP inhibits platelet aggregation in washed platelets and in platelets that were reconstituted with defibrinogenated plasma. Thus, the inhibition of platelet aggregation by proteinase F1 is independent of its action on fibrinogen [78]. We speculated that the inhibition could be due to either binding to or hydrolysis of a plasma factor, or to accumulation of inhibitory peptides formed during the hydrolysis of a plasma factor other than fibrinogen.

In 1992, Huang et al. purified a P-I SVMP from *Agkistrodon rhodostoma* (=*Calloselsma rhodostoma*) venom that inhibited platelet aggregation [79]. It inhibited aggregation induced by low concentrations of thrombin (≤0.2 U/mL) with only slight effect on aggregation induced by high concentrations of thrombin (≥0.5 U/mL) [80]. This enzyme, named Kistomin, significantly inhibited cytosolic Ca^2+^ rise, completely blocked formation of thromboxane B2 and inositol phosphates in platelets stimulated by 0.1 U/mL of thrombin. In contrast, it inhibited significantly thromboxane but not inositol phosphates formation of platelets stimulated by a high concentration of thrombin (1 U/mL). They showed that incubation of platelets with kistomin resulted in a selective cleavage of platelet membrane glycoprotein Ib (GPIb) [80]. These results suggested that (a) kistomin is a highly selective SVMP that cleaves GPIb; and (b) thrombin activates platelets at least through two receptors; GPIb and a second receptor. Intact GPIb plays critical role in the extent and rate of platelet aggregation stimulated by low concentrations of thrombin [80]. Kistomin cleaves platelet GPIbα at two distinct sites releasing 45- and 130-kDa soluble fragments and specifically inhibits von Willebrand factor- (vWF-) induced platelet aggregation [81]. Kistomin also cleaves vWF resulting in the formation of low-molecular-mass multimers. It inhibits GPIbα agonist-induced platelet aggregation, and prolongs the occlusion time in mesenteric microvessels and tail-bleeding time in mice [81]. Kistomin also inhibits platelet aggregation induced by collagen and convulxin (Glycoprotein VI (GPVI) [82]. It cleaves GPVI but not integrins α_2_β_1_ and α_IIb_β_3_. The release of 25- and 35-kDa fragments from GPVI suggests that kistomin cleaved GPVI near the mucin-like region. Hsu et al. identified that kistomin cleaves Glu_205_-Ala_206_ and Val_218_-Phe_219_ peptide bonds using synthetic peptides [82]. Thus, P-I SVMP kistomin specifically targets receptors GPIbα and GPVI on platelets and vWF in the plasma to exhibit its effects on platelet aggregation. Kistomin may be useful for studying metalloprotease-substrate interactions and has a potential being developed as an antithrombotic agent. Huang and colleagues also characterized crotalin, a P-I SVMP from venom of *Crotalus atrox* that also cleaves vWF and GPIbα [83].

Mocarhagin, a 55-kDa SVMP from *Naja mocambique mocambique* (=*Naja mossabica*) cleaves GPIbα [84]. The GPIbα fragment cleaved by this SVMP, His_1_-Glu_282_ was useful in identifying the thrombin-binding site; the sulfated tyrosine/anionic segment Y_276_DYYPEE_282_ are important for the binding of thrombin and the botrocetin-dependent binding of vWF [84]. Interestingly, mocarhagin cleaves a 10-amino acid residue peptide from the N-terminus of P-selectin glycoprotein ligand receptor (PSGL-1) expressed on neutrophils to abolish P-selectin binding on endothelial cells and prevents rolling of neutrophils [85]. In both cases, mocarhagin targets mucin-like substrates (GPIbα and PSGL-1) within anionic amino acid sequences containing sulfated tyrosines. Brendt and colleagues showed the presence of SVMPs that are immunologically and functionally similar to mocarhagin in *N. kaouthia* (*N. siamensis*), *N. nivea* (*N*. flava), *N. nigricollis crawshawii*, *N. nigricollis pallida*, *N. nigricollis nigricollis*, *N. atra*, *N. haje*, *N. naja*, *N. melanoleuca* and *N. oxiana*, but not in *N. sputatrix* venoms [86]. They also developed a simple method for purification of SVMPs using Ni^2+^-agarose column and purified Nk from *Naja kaouthia* venom that cleaves GPIbα [87]. During the subsequent studies, same group found out that nerve growth factor (NGF) binds to Ni^2+^-agarose column and NGF is co-purified with SVMPs [88]. They showed venom NGF and human NGF inhibits both SVMPs and human MPs.

Interestingly, another distinct P-III SVMP, NN-PF3, that inhibits platelet aggregation was purified and characterized from *Naja naja* venom [89]. NN-PF3, unlike the above *Naja* SVMPs, fails to inhibit ristocetin-induced platelet aggregation. Instead, it inhibits collagen-induced aggregation of washed platelets [89]. Western blot using anti-integrin α_2_β_1_ mAb 6F1 suggested that NN-PF3 binds to α_2_β_1_ integrin in a sequence-dependent manner only but does not cleave α_2_β_1_ integrin. However, there is a drastic reduction in several intracellular signaling [89]. Further mechanistic details and structure–function relationships of NN-PF3 may help delineate the differences in the targeting of *Naja* SVMPs.

Jararhagin from *Bothrops jararaca* (Brazilian pit-viper) venom is a P-III SVMP with MDC domains [90]. The RGD tripeptide sequence in the D domain is replaced by ECD sequence. Jararhagin cleaves the C-terminal part of fibrinogen Aα chains, resulting in the removal of a 23 kDa fragment while leaving the β and γ chains unaffected [91]. The cleaved fibrinogen molecule is still fully functional in both platelet aggregation responses to ADP and adrenalin and in its ability to clot plasma by thrombin. However, the fibrin polymerization is abnormal [91]. Jararhagin inhibits both ristocetin- and collagen-induced platelet aggregations. The inhibition of ristocetin-induced platelet aggregation is attributed to a direct cleavage of vWF rather than its receptor GPIb-IX-V [92]. The cleavage vWF occurs in the N-terminal half, which contains the binding site for the GPIb receptor, the AI domain. Hydrolysis of vWF leads to the disappearance of the high molecular size multimeric structure of vWF and loss of platelet responses [92]. Ivaska et al. designed a series of eight short cyclic peptides corresponding to hydrophilic and charged regions along the protein sequence to identify the α2I binding site [93]. The peptide spanning C*_241_TRKKHDNAQ_249_C* (*Cys residues form the disulfide bond) binds to α2I domain and interferes with the interaction between α2I domain and collagen. Using Ala scanning method, they identified the importance of RKK tripeptide sequence for this interaction [93]. Finally they developed a shorter, more potent version of this peptide C*TRKKHDC* which inhibits α2I domain and collagen interaction with an IC_50_ of 1.3 mM. These peptides bind near the metal ion-dependent adhesion site of the human integrin α_2_I-domain [94]. The peptide C*TRKKHDC* competes for the collagen-binding site of α_2_I but does not induce a large scale conformational rearrangement of the I domain [95].

In contrast, the inhibition of collagen-induced aggregation is driven by interference with the α_2_β_1_ integrin, but not GPVI receptor [96]. However, treatment of platelets with jararhagin drastically reduces α_2_β_1_ integrin on the platelet surface [92,97]. The effect was attributed both to binding to the α_2_I domain [97] and to cleavage of the α_2_β_1_ integrin [92,98]. The degradation of the β_1_ subunit of α_2_β_1_ by jararhagin results in the loss of pp72^syk^ phosphorylation and thus β_1_ subunit appears to be critically involved in collagen-induced platelet signaling [99]. Using recombinant fragments and monoclonal antibodies, Tanjoni et al. showed that jararhagin binding to collagen and α_2_β_1_ integrin occurs by two independent motifs, which are located on D and C domains, respectively [99]. The roles of non-enzymatic domains in platelet aggregation are discussed below.

In addition to jararhagin (described above), several other P-III SVMPS, such as atrolysin A from *Crotalus atrox* venom [100], catrocollastatin from *Crotalus atrox* venom [101], crovidisin from *Crotalus viridis* venom [102], alternagin from *Bothrops alternatus* venom [103], acurhagin from *Agkistrodon acutus* venom [104], halydin (D domain from a P-III) from *Gloydius halys* venom [105] and kaouthiagin from *Naja kaouthia* venom [106] inhibit collagen-induced platelet aggregation. Mechanistically, these SVMPs bind and/or proteolytically cleave vWF, collagen, GPVI or α_2_β_1_. Interestingly, acurhagin (87% identity with jararhagin) selectively inhibits platelet aggregation induced by collagen and suppresses tyrosine phosphorylation of several signaling proteins in convulxin-stimulated platelets [104]. Thus, acurhagin exhibits its function mainly through its binding to GPVI and collagen, instead of binding to α_2_β_1_, or cleaving platelet membrane glycoproteins [104]. Recently, a P-I SVMP from *Bothrops barnetti* venom that inhibits platelet aggregation induced by vWF *plus* ristocetin and collagen was characterized [107]. It presumably cleaves both vWF and GPIb and thus, inhibits vWF-induced platelet aggregation. It also cleaves the collagen-binding α_2_A domain of α_2_β_1_ integrin and thus, inhibits collagen-induced platelet aggregation [107]. Despite the missing D and C domains, this P-I SVMP has similar properties compared jararhagin, a P-III SVMP. Such examples will help us understand subtleties in structure–function relationships.

## 5. Platelet Aggregation Agonists

A small number of SVMPs have been shown to induce platelet aggregation. Alborhagin, a P-III SVMP isolated from *Trimeresurus albolabris* venom activates platelets through a mechanism involving GPVI [108]. It induces similar tyrosine phosphorylation pattern [108] to convulxin, a GPVI agonist [109,110,111]. Interestingly, alborhagin has minimal effect on convulxin binding to GPVI-expressing cells, suggesting that these proteins may recognize distinct binding sites on GPVI. Both alborhagin and crotarhagin from *Crotalus horridus horridus* venom induce platelet aggregation [112]. They induce ectodomain shedding of GPVI by a mechanism that involves activation of endogenous platelet metalloproteases. This shedding of 55-kDa soluble GPVI fragment required GPVI-dependent platelet activation [112].

## 6. Role of Non-Enzymatic Domains and Subunits

In snake venoms, three distinct classes of SVMPs, P-I, P-II and P-III, are produced [3,4]. These enzymes exhibit various pharmacological effects by binding to specific target proteins. In most cases, the cleavage of the target proteins through their Zn^2+^-dependent proteolytic activity leads to either destruction of the receptor or release of new ligands. Thus, M domain plays critical role in most of the pharmacological activities exhibited by SVMPs. However, in a significant number of instances, SVMPs exhibit their functions by non-enzymatic mechanisms through selective binding to key proteins. In such cases, non-enzymatic domains, such as D and C domains, as well as non-enzymatic subunits, such as snaclecs, play important roles. At times, these domains are proteolytically “processed” and exhibit independent pharmacological effects [3,4,113]. It is important to note that in some cases proteolytic activity is essential for the biological effects, while in others just physical binding and steric interference is sufficient for the function (although cleavage may still occur in any case). In this section, we will highlight the roles of these non-enzymatic domains and subunits in specific binding to the target proteins and inducing pharmacological effects.

As mentioned above, precursor of SVMPs are “processed” into various proteolytic products [113,114]. Accordingly, “processing” of P-II SVMPs lead to separation of M and D domains (P-I-like SVMPs and disntegrins, respectively), while “processing” of P-III SVMPs lead to separation of M and DC domains. In 1994, Usami et al. isolated jararhagin-C, a 28 kDa protein containing the DC domain of jararhagin [115]. Jararhagin-C inhibits collagen- and ADP-induced platelet aggregation in high nanomolar concentrations [115]. Interestingly, phenanthroline-inactivated jararhagin inhibits collagen-induced platelet-aggregation with similar potency [96]. Native jararhagin is only 3- to 4-times more active than inactive jararhagin. These results suggest that there is a significant contribution of non-enzymatic mechanism to the inhibition of platelet aggregation and the small difference is due to proteolytic activity (enzymatic component) of jararhagin. Similarly, native and recombinant DC domains of alternagin, catrocollastatin and atrolysin A inhibit collagen-induced platelet aggregation [100,103,116]. In contrast, leberagin-C, DC domain containing protein from *Macrovipera labetina transmediterranea* venom inhibits platelet aggregation induced by thrombin and arachidonic acid with IC_50_ of 40 and 50 nM, respectively [117]. It inhibits the adhesion of melanoma tumor cells on fibrinogen and fibronectin, by interfering with the function of α_v_β_3_ and, to a lesser extent, with α_v_β_6_ and α_v_β_1_ integrins. It does not bind to α_2_β_1_ integrin. These studies support the importance of DC domains in the inhibition of platelet aggregation through non-enzymatic mechanisms. Structure–function relationships of these DC domains will help in determining the integrin selectivity and binding.

As with DC domains, “processed” D domains were also isolated as disintegrins from crotalid and viperid venoms. Disintegrins are among the potent inhibitors of platelet aggregation peptides [118,119,120,121,122,123,124]. These polypeptides, ranging from 49 to 84 amino acid residues, are isolated from crotalid and viperid snake venoms. They have a RGD/KGD tripeptide sequence in a 13-residue β-loop structure (dubbed as RGD loop), which is responsible for their biological activity. The active tripeptide RGD is located at the apex of a mobile loop protruding 14–17 Å from the protein core [125,126] and plays key role in the interaction of the disintegrins with the platelet integrin α_IIb_β_3_ [127,128]. These disintegrins are derived by the processing of the D domains from P-II SVMP precursors [113]. Disintegrins with RGD sequence show different levels of binding affinity and selectivity towards α_IIb_β_3_, α_v_β_3_ and α_5_β_1_ integrins [129], while KGD-containing barbourin inhibits the α_IIb_β_3_ integrin with a high degree of selectivity [130]. This RGD tripeptide is replaced by various sequences including VGD, MLD, MVD and KTS, resulting in distinct integrin selectivity [131] and references therein]. Despite the role of disintegrins in inhibiting platelet aggregation, we will not focus on this group of non-enzymatic polypeptides. Readers can obtain details on this group of fascinating molecules elsewhere [132,133,134,135,136,137].

In significant number of P-III SVMPs, RGD sequence is replaced by various tripeptide sequences (for example, see [131]) and at the apex of the loop is a Cys residue involved in forming a disulfide bond. Thus, this D domain is appropriately named as “disintegrin-like” domain [3,4]. As with disintegrins, “disintegrin-like” D domains play important role in the recognition of various target receptors or integrins and inhibit platelet aggregation [100,106,138,139,140]. Recombinantly expressed D domain of jararhagin inhibits platelet–collagen interaction [140]. Linear peptides based on this domain were shown to inhibit the release of 5-hydroxytryptamine (5-HT) from collagen-stimulated platelets [140]. This selective inhibition of the secretion-dependent phase by jararhagin and its peptides is due to the defective phosphorylation of pleckstrin, which is involved in dense granule secretion [140]. Cyclic peptides that cover the loop inhibit platelet aggregation [139] as well as bind to collagen [93,95]. These studies indicate that the non-enzymatic D domains indeed plays critical role in recognition and binding of target receptor or integrin.

Thus far, polypeptides containing only C domain have not been isolated from snake venoms. Therefore, C domains are recombinantly expressed to evaluate their role in platelet functions. C domain of atrolysin A potently inhibits collagen- but not ADP-stimulated platelet aggregation [141]. These studies suggested that the C domain interacts with the collagen receptor α_2_β_1_ integrin on the platelet surface. Using overlapping peptides from C domains of atrolysin A and jararhagin, Kamiguti et al. identified two peptides each corresponding to identical segments [142]. These peptides inhibit collagen-induced aggregation, but not convulxin-induced. Thus, they interact with α_2_β_1_ integrin and not through GPVI. VKC-jararaca, but not VKC-atrox, induced a rapidly reversible weak aggregation [142]. Pinto et al. identified two regions, _365_PCAPEDVKCG_374_ and _372_KCGRLYCK_379_ in C domain of jararhagin which could bind to vWF [143]. They ruled out the latter region using molecular modeling and docking experiments. The C domain of atrolysin A not only bound directly to vWF and collagen I, but also blocked the collagen–vWF interaction [144]. The interaction of the C domain with the A1 domain of vWF promotes vWF proteolysis and inhibition of vWF-mediated platelet aggregation [145,146]. Similarly, C domain plays crucial role in ADAMTS-13, a vWF-cleaving protease; removal of this domain leads to a remarkable reduction of its ability to cleave vWF [147]. These studies strongly support the importance of the C domain in the non-enzymatic mechanism of inhibition of platelet aggregation.

Thus far, only snaclecs are found to be associated with P-III SVMPs [10,11,12,47,58]. As discussed above, these subunits are covalently linked through P-III SVMPs by interchain disulfide bond. As with other snaclecs, these subunits are heterodimeric proteins with two chains linked by an interchain disulfide bond. The concave dimeric interface forms the ligand-binding site of FX and prothrombin [17,47,56,58]. Respective Gla domains bind to these subunits in a Ca^2+^-dependent manner and provide excellent selectivity. Thus, these non-enzymatic subunits impart to distinct properties. Correctly modified and folded Gla domain is important for optimal activity. It defines the Ca^2+^-dependence, as Ca^2+^ ions are required for proper folding of Gla domain. Carinactivase-1 and multactivase fail to activate prethrombin-1 and descarboxyprothrombin in which Ca^2+^-binding has been perturbed. On the other hand, Ecarin, which does not have this subunit, activates prothrombin, prethrombin-1 and descarboxyprothrombin with equal efficiency. This functional difference helps in measuring normal prothrombin versus descarboxyprothrombin in the plasma of warfarin-treated individuals [57]. Thus, these non-enzymatic regulatory subunits play critical role in substrate recognition and selectivity.

## 7. Definition and Nomenclature for Interaction Sites in Proteases

Proteases recognize and interact with specific substrates by binding them through various functional residues distributed among different sites. Each of these sites plays a specific role in the overall function of the enzyme. Our understanding of the chemical and biophysical interactions of various substrates with their respective enzymes has helped us to define these sites. Based on the interactions of SVMPs with various substrates, receptors and integrins (discussed above), we would like to propose new definitions of additional functional sites. We will also provide distinguishing features of these new sites in comparison with established functional sites.

### 7.1. Active Site

It is the region where substrate molecules bind (binding site) and undergo a chemical reaction (catalytic site). Binding site correctly orients the substrate for catalysis, while residues in the catalytic site play mechanistic role in lowering the activation energy to make the reaction proceed faster. Specific amino acid residues, cofactors and/or ions play critical roles in the catalytic mechanisms in protein enzymes. For example, each residue in the catalytic triad (Ser, His and Asp/Glu) plays a role in catalysis in serine proteases. The Acid–Base–Nucleophile triad generates a nucleophilic residue for covalent catalysis [148]. The residues form a charge-relay network to polarize and activate the nucleophile, which attacks the substrate and forms a covalent intermediate, which is then hydrolyzed to regenerate free enzyme. The nucleophile in serine proteases is a Ser; Cys, and occasionally Thr, also serve as nucleophile in other classes of proteases. Catalytic cleavage in SVMPs is through Zn^2+^ coordinated by three conserved His side chains and a water anchored to a conserved Glu [24,25]. This polarized water molecule acts as general base that catalyzes peptide bond cleavage.

Substrate binding site can be quite elaborate and complex; higher the complexity better is the substrate selectivity. The substrate binding site is divided into several subsites—the regions, which are on the enzyme surface that interact with individual amino acid residues on either side of the substrate cleavage site. The subsites on the amino side of the cleavage site are labeled as S1, S2, S3, etc. (non-prime subsites), while those on the carboxyl side are labeled as S1′, S2′, S3′, etc. (prime subsites). Generally, these are discontinuous sites and thus, the residues forming these subsites are not contiguous in the protein sequence. P1 amino acid residue of the substrate associates with S1, P2 with S2, etc. Similarly, P1′ amino acid residue binds to S1′, P2′ with S2′, etc. P1-P1′ peptide bond of the substrate is proteolytically cleaved. Both non-prime and prime subsites could contribute to substrate selectivity and affinity. Paes Leme et al. [149] determined the amino acid preferences across the full P4 to P4′ range for the three P-I SVMPs, leucurolysin-a, atrolysin C, and BaP1, and one P-III SVMP, bothropasin, using high resolution mass spectrometric method and albumin-depleted plasma tryptic peptide library. All these SVMPs showed preferences (clear specificities) towards large, hydrophobic aliphatic residues at P1′, P2′ and P3′ sites [149].

### 7.2. Exosite

This is a secondary binding site, remote from the active site, on the enzyme. Exosites provide additional substrate (or inhibitor) selectivity. For example, thrombin (a serine protease) has two distinct electropositive surface regions, exosite I and exosite II, that contribute to the specificity of thrombin [150,151]. These exosites mediate the interactions of thrombin with its substrates, inhibitors and receptors. Exosite I is adjacent to the P′ side of the active site cleft and is the fibrinogen recognition exosite. Exosite II is more basic than exosite I and it binds to heparin. For details on the interaction of these exosites with substrates, receptors and inhibitors, see [150,151,152]. In SVMPs there is an exosite C*_241_TRKKHD_246_C* (as numbered in jararhagin) that interacts with human integrin α_2_I-domain [93,94,95]. Because of their importance in determining exquisite selectivity and specificity, the exosites are of immense interest in biomedical research as potential drug targets [153,154,155,156,157,158].

### 7.3. Allosteric Site

Small regulatory molecules interact with this site on the enzyme to activate or inhibit (positive or negative allosterism) the specific enzyme. In general, the non-covalent and reversible interaction of the allosteric effector often results in a conformational change. In homotropic allosterism, the modulator molecule is the substrate as well as the regulatory molecule for the target enzyme. It is typically an activator of the enzyme. In contrast, in heterotropic allosterism, modulator is not the enzyme’s substrate. In this case, the modulator may be either an activator or an inhibitor. Although multimeric proteins (e.g., hemoglobin and ATPase) are considered to be prone to allosteric regulation, even monomeric proteins (e.g., myoglobin, human serum albumin, and human α-thrombin) exhibit heterotropic allosterism [159,160,161]. The rational design of specific antagonists targeting the active site to highly homologous enzymes is an extremely difficult task. As with exosites, allosteric sites are also used for designing drugs targeting specific enzymes [162,163]. For details on protein allosteric mechanisms, see [164].

### 7.4. Exosite versus Allosteric Site

Both exosite and allosteric site are on the surface of the enzyme or receptor. In the case of exosite, one part of the substrate or inhibitor interacts with the exosite while the other part interacts with the active site. Thus, exosite typically must be occupied first for optimal activity. In contrast, a substrate molecule (homotropic allosterism) or a ligand (heterotropic allosterism) interacts with the allosteric site and a second substrate molecule interacts with the active site. The binding at the allosteric site enhances or decreases the binding or catalysis at the active site. Thus far, no allosterism has been documented in SVMPs.

### 7.5. Classification of Exosites and Allosteric Sites (Figure 4)

**Figure 4:**
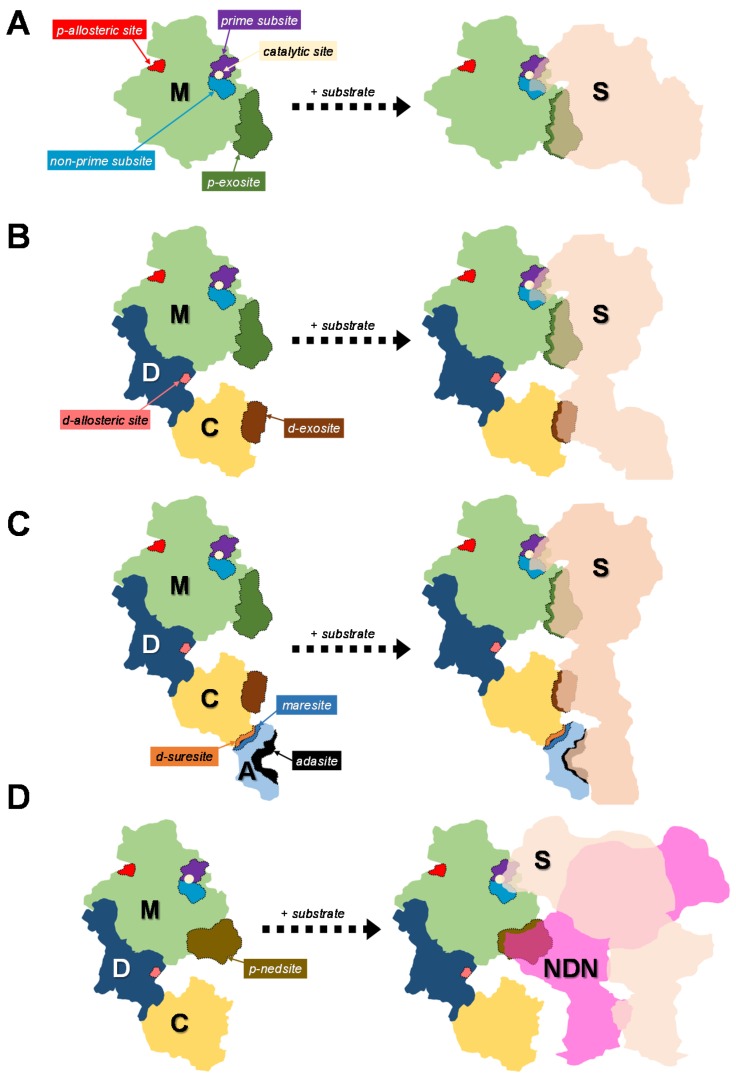
Nomenclature of interaction sites in snake venom metalloproteases. Left and right columns show free and respective substrate-bound protease. (**A**) **Left**: M domain showing catalytic site, prime and non-prime subsites, and proximal allosteric and exosites. **Right**: Substrate S interacts with the protease through active site and p-exosite. (**B**) **Left**: MDC domains showing distal allosteric and exosites. **Right**: Substrate S interacts with the protease through active site, p-exosite and d-exosite. (**C**) **Left**: MDC domains showing distal suresite and adaptor subunit, A showing interaction with MDC domain through distal maresite. A subunit also shows adasite. **Right**: Substrate S interacts with the protease through active site, p-exosite, d-exosite and adasite. (**D**) **Left**: MDC domains showing proximal nedsite. **Right**: Next-door neighbor (NDN) subunit interacts with p-nedsite, while the substrate S interacts with the protease through active site. See text for details.

Exosites and/or allosteric sites can be present in the same domain as the orthosteric site, such as active site (in enzymes) or agonist binding site (in receptors). These sites are thus closer to the orthosteric site and located on the enzymatic M domain and we name them as “p-exosite” (proximal-exosite) and “p-allosteric site” (proximal-allosteric site) (Figure 4A,B). The examples of p-exosites are exosite I and exosite II of thrombin [150,151] and C*_241_TRKKHD_246_C* exosite of SVMPs [93,94,95]. In multi-domain enzymes and receptors, these regulatory sites may also be found in other domains. In such cases, we name them as “d-exosite” (distal-exosite) and “d-allosteric site” (distal-allosteric site) (Figure 4A,B). It is possible that these distal sites residing in different domains may be located physically closer to the orthosteric site in the tertiary structure of the proteins. The sites on D and C domains of SVMPs are excellent examples of d-exosites. A better understanding of the distance between orthrosteric site and the regulatory sites will be helpful in designing bifunctional ligands for the target enzyme or receptor.

Enzyme complexes, such as RVV-X, carinactivase-1 and multactivase [38,47,58], are heterodimers comprising a larger main subunit and smaller snaclec subunits. These enzymes use the concave dimeric interface of the snaclec subunits to bind to the substrate [8,47,58]. If these snaclec subunits of these SVMPs or the Gla domains of the substrates are removed, the substrate interaction is extremely poor. Thus, the concave dimeric interface of the adapted subunit acts as the exosite. Therefore, we named this site as “adasite” (adaptor exosite) (Figure 4C). In these cases, there are mutual recognition sites that form the interface between the SVMP and the snaclec subunit. These interaction sites are named as “maresite” (main subunit recognition site on the smaller subunit) and “suresite” (smaller subunit recognition site on the main subunit) (Figure 4C). As with exosite and allosteric sites, suresites can be either “p-suresite” (proximal-suresite, when located on the enzymatic M domain) or “d-suresite” (distal-suresite, when located in other domains). The finer definition and differentiation among various interaction sites will help improving the clarity in the field of SVMPs as well as other enzymes and receptors.

## 8. Unusual Behavior of Metalloproteases

During our analyses of the literature, we found two interesting, somewhat unusual behaviors of SVMPs. We have highlighted these observations as they will be useful in future research strategies in the field of SVMPs as well as other proteases.

### 8.1. Binding to Cell Surface Receptors

**Figure 5:**
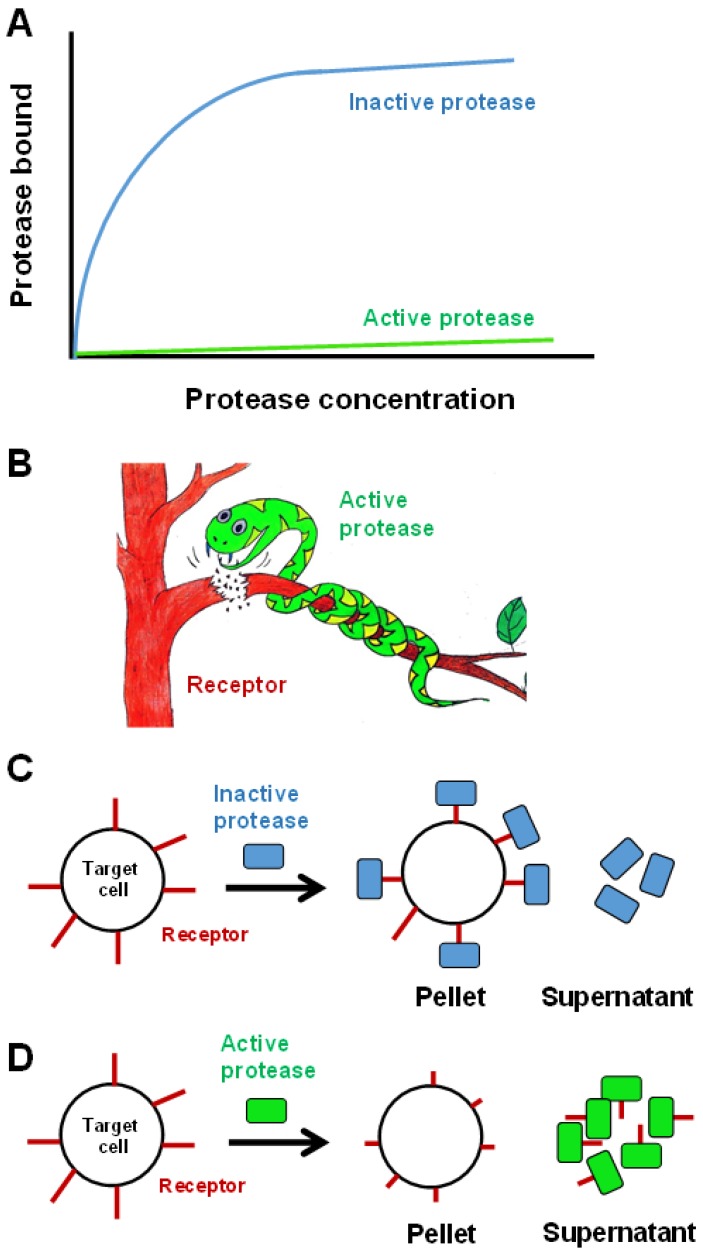
Unusual specific binding of snake venom metalloproteases with target receptor. (**A**) Schematic diagram showing specific binding of active and inactive SVMPs. Diagram is drawn based on the data published by Kamiguti et al. [96]. (**B**) Active protease cleaves the receptor and gets released into the solution. The picture was created by Cho Yeow Koh and Pol Zen Koh. (**C**) Inactive protease binds to receptors on the surface of the target cells and remains bound to the cells in the precipitate. (**D**) Active protease, on the other hand, cleaves the receptor and remains in the supernatant indicating low or no binding to cells in the precipitate.

A key step in the identification of target receptor or acceptor on the cell surface is the characterization of specific binding and Scatchard plots [165,166]. Kamiguti et al. performed binding studies using ^125^I-jararhagin to determine specific binding to platelets [96]. Their experiments showed no significant specific binding. Intelligently, they also studied the equilibrium binding of 1,10-phenanthroline-treated, catalytically inactive ^125^I-jararhagin to platelets. The inactive jararhagin showed excellent specific binding to platelets (Figure 5). They had earlier determined that treatment of platelets with jararhagin drastically reduces α_2_β_1_ integrin on the platelet surface [92,96]. These observations can be easily explained by the binding of active jararhagin to α_2_β_1_ integrin and subsequent cleavage leading to the release of jararhagin from the platelet surface (Figure 5C,D). In contrast, inactive jararhagin continued to bind to α_2_β_1_ integrin and stay bound to the platelet surface in the absence of proteolytic activity. Thus, the diligent strategy used by Kamiguti et al. makes an important contribution to specific binding studies of SVMPs. These strategies will also be extremely useful in studying specific binding of other proteases to cell surface receptors.

### 8.2. Unusual Cleavage of the α_2_β_1_ Integrin

In general, proteases bind to a protein substrate and then cleave one or more peptide bonds of this substrate. Jararhagin and other SVMPs have an unusual behavior in cleavage of the α_2_β_1_ integrin. They bind to α_2_I domain of the α_2_ integrin and cleave the β_1_ subunit [97,98]. Thus, the binding and cleavage occur in two distinct protein subunits; these SVMPs bind to one protein subunit, but cleave the “next door neighbor” subunit. Such proteolytic cleavage away from the vicinity of the binding site may not be uncommon. The functional exosite that facilitates cleavage in the neighboring protein is named as “nedsite” (next door site) (Figure 4D). Nedsite can be further classified as either “p-nedsite” (proximal-nedsite, when located on the enzymatic M domain) or “d-nedsite” (distal-nedsite, when located in other domains). The p-exosite C*_241_TRKKHD_246_C* of SVMPs [93,94,95] that binds to α_2_I domain should be properly identified as a p-nedsite.

## 9. Anticoagulant and Antiplatelet Activity in Hemorrhage

SVMPs frequently induce hemorrhage through the degradation of matrix proteins and basement membrane, resulting in the disruption of endothelial cell integrity in blood vessel walls [143,167,168,169]. This extra-vascular blood leakage is exacerbated by the disturbance of blood coagulation and platelet aggregation. A number of snake venom toxins have evolved to target various points along the blood coagulation cascade and platelet aggregation pathways. These toxins exhibit both pro- and anti-coagulation of blood or pro- and anti-platelet aggregation effects. Procoagulant toxins not only activate factor VII, factor X, factor V, and prothrombin but also act directly on fibrinogen [170,171]. In the whole animal, defibrogenating the blood and removing significant number of blood coagulation protein result in unclottable blood through consumptive coagulopathy [172]. In addition, a number of SVMPs interfere in blood coagulation and platelet aggregation (described above) and thus enhance hemorrhage. For example, Jararhagin affects hemostasis through fibrinogen degradation [91,173] and by the inhibition of platelet aggregation [92]. These effects significantly enhance its own as well as venom’s hemorrhagic activity.

## 10. SVMPs as Research Tools, and Diagnostic and Therapeutic Agents

Due to high specificity and selectivity, SVMPs and their parts are used in various applications. Among them, their uses as diagnostic agents in hematology laboratories are well known. Stypven (Styptic venom) time is one the earliest one-step clotting time [174]. Russell’s viper venom (capable of stopping the bleeding when applied to a wound and hence styptic venom) activates FX directly to initiate coagulation. The Stypven time is unaffected by deficiencies or abnormalities of factors VII, XII, XI, IX or VIII. However, it is abnormal in FV, prothrombin and in most cases of FX deficiency. Thus, it is used to detect hereditary deficiencies or abnormalities and disease- or drug-induced deficiencies. A modified version with limiting amounts of phospholipid and venom, dilute Russell viper venom time, is used for the detection of lupus anticoagulants [175,176]. The individuals with a lupus anticoagulant produce autoantibodies that bind to phospholipids. These antibodies prolong the clotting time by binding to phospholipids in dilute Russell viper venom time, a simple, reproducible, sensitive, and relatively specific method. The ecarin clotting time (ECT) allows us to carry out precise quantification of direct thrombin inhibitors [177]. Ecarin [49], a specific prothrombin activator, activates prothrombin to generate meizothrombin. The cleavage of a chromogenic substrate by meizothrombin is inhibited by direct thrombin inhibitors in a concentration-dependent fashion [178]. Various modifications of the ECT are important in both preclinical and clinical use, e.g., for biochemical investigations, as a point-of-care method and for cardiac surgery. For details of the advantages and disadvantages of these methods, see [177,178]. In CA-1 method, carinactivase-1 [47], a Ca^2+^-dependent prothrombin activator, is used to activate prothrombin [57]. Since carinactivase-1 recognizes the carboxylated, fully folded Gla domain of prothrombin, CA-1 method measures only normal prothrombin and not descarboxyprothrombin (produced in warfarin-treated individuals). Thus, CA-1 method is a novel assay for monitoring coagulant activity in warfarin-treated individuals. For details on other snake venom proteins used as diagnostic agents, see [179,180].

SVMPs and their domains have also significantly contributed as research tools and also in the development of therapeutic leads. Although classical snake venom D and DC domains are proteolytically released from PII and PIII SVMPs [4,113,114], some heterodimeric disintegrins are encoded by separate genes [181,182,183]. Most common disintegrins with RGD motif bind selectively with high affinity to integrins including fibrinogen receptors (α_IIb_β_3_), vitronectin receptors (α_v_β_3_) and fibronectin receptor (α_5_β_1_). Disintegrins with MLD motif are heterodimeric disintegrins and bind to α_4_β_1_, α_4_β_7_ and α_9_β_1_ integrins. When their second subunit contains RGD, they bind to α_5_β_1_ integrin [184]. Disintegrins with KTS/RTS motif bind to α_1_β_1_ integrin [135,185,186,187,188,189]. The selectivity and potency strongly depends on the amino acid composition surrounding RGD/MLD/KTS/RTS motifs. For details on the selectivity of various disintegrins, see [134,136,137,184,186,190,191]. DC domains have a limited anti-integrin activity. Alternagin-C binds to collagen receptor, α_2_β_1_ integrin through its RSECD sequence located in the D domain [102]. Leberagin-C binds to α_v_β_3_ integrin [117]. However, specific integrin-binding motif was not evaluated. Because of their highly specific and selective interaction with various integrins, these disintegrins modulate cellular responses in platelets, neutrophils, T-lymphocytes, eosinophils and endothelial cells as well as various cancer cells (for details, see [134,137,190] and references therein). In addition, they also exhibit uniquely exclusive effects on smooth muscle cells [191,192], fibroblast-like cells [193,194], chondrocytes [195], osteoblasts [196], and neuronal progenitors [197]. Recent studies using obtustatin has shown that α_1_β_1_ integrin and integrin-linked kinase modulate angiotensin II effects in vascular smooth muscle cells and thus, are potential targets to the development of more effective therapeutic interventions in cardiovascular diseases [198,199]. Thus, D and C domains selectively target specific integrins and play critical role in our understanding of cell biology.

The high specificity, selectivity and affinity of D domains have helped the scientific community to design potent therapeutic agents for various human diseases. For example, RGD-disintegrins resulted in the successful design of two therapeutic drugs that inhibit α_IIb_β_3_ integrin and are approved for the treatment of acute coronary ischemic disease and prevention of thrombotic complication in balloon angioplasty and stenting [200,201]. Integrilin (Eptifibatide, a synthetic cyclic heptapeptide) and tirofiban (Aggrastat, a non-peptide RGD mimic) were designed based on the structure of barbourin [121] and echistatin [119], respectively. Native or recombinant contortrostatin, a homodimeric RGD-disintegrin from *Agkistrodon contortrix contortrix* venom, exhibits potent antiangiogenic effects in in vitro and in vivo models [202,203,204]. Using liposomal delivery is effective as an anti-tumor agent in animal models of human breast, ovarian and prostate cancer [204,205]. A chimeric variant, Vicrostatin induces apoptosis and blocks tube formation in Matrigel [206]. Based on KTS-disintegrins, Vimocin and Vidapin (cyclic KTS peptides) that target α_1_β_1_/α_2_β_1_ integrins are being developed as potent antagonists of angiogenesis for the treatment of angiogenesis disorders and cancer [207], whereas Vipegitide and Vipegitide-PEG2 (peptidomimetics) that target α_2_β_1_ integrin are being developed as another class of inhibitors of platelet aggregation for antithrombotic therapy [208]. Thus, research on the non-enzymatic D and C domains, which interact with integrins, have contributed significantly and appear to have tremendous future in basic cell biology as well as in biomedical applications [133,137,183,209,210].

SVMPs and their catalytically active M domains are also important in the development of therapeutic agents. A direct fibrinolytic enzyme from *Agkistrodon contortrix contortrix* venom, fibrolase and its recombinant analog, alfimeprase was developed as a clot-buster drug for myocardial infarction and stroke due to its thrombolytic properties [67,211,212,213]. Alfimeprase reached Phase 1 and Phase 2 clinical trials [214,215], but did not make it to the market. For details, see [216]. Despite the setback, there are several lessons learnt through their efforts. Dual antithrombotic therapy using hirudin (thrombin inhibitor) and S18886 (thromboxane A_2_ receptor antagonist) were shown to improve reperfusion after thrombolysis with alfimeprase but not tissue plasminogen activator [217]. A careful strategy may help in developing this and related SVMPs as an alternative thrombolytic agent (clot buster) in clearing cardiovascular and cerebrovascular blockages in myocardial infarction and stroke.

## 11. Summary and Future Prospects

SVMPs, and their domains and complexes have evolved to bind to various integrins, receptors and extracellular matrix proteins. They activate or inactivate proteins through enzymatic or non-enzymatic mechanisms and interfere in blood coagulation and platelet aggregation, and contribute to venom toxicity, particularly to hemorrhagic activity and venom distribution in the prey or victim. The understanding of their structure–function relationships and mechanism of action has contributed significantly to basic sciences including protein chemistry, enzymology, hematology, angiogenesis and cancer biology, and also helped in the development of diagnostic and therapeutic agents. Further studies on this group of toxins will contribute to unlocking several complex physiological processes and pathological effects in blood coagulation, platelet aggregation, hemorrhage, matrix biology, angiogenesis and cancer biology. Their structure–function studies will enhance the potential in developing novel diagnostic and therapeutic agents.
